# Correlation analysis of physical fitness and its impact on falls in 2130 community- dwelling older adults: a retrospective cross-sectional study

**DOI:** 10.1186/s12877-022-03138-9

**Published:** 2022-05-23

**Authors:** Wang-Sheng Lin, Nai-Wei Hsu, Meng-Jer Lee, You-Yuan Lin, Chih-Chun Tsai, Po-Jung Pan

**Affiliations:** 1 Department of Physical Medicine & Rehabilitation, Taipei Veterans General Hospital, Yuan-Shan/Su-Ao Branch, Yilan, Taiwan; 2 School of Medicine, National Yang Ming Chiao Tung University, Taipei, Taiwan; 3 Public Health Bureau, Yilan County, Taiwan; 4 Community Medicine Research Center & Institute of Public Health, School of Medicine, National Yang Ming Chiao Tung University, Taipei, Taiwan; 5 Department of Physical Medicine & Rehabilitation, National Yang Ming Chiao Tung University Hospital, Yilan, Taiwan; 6 Department of Mathematics, Tamkang University, Taipei, Taiwan; 7 Center of Community Medicine, National Yang Ming Chiao Tung University Hospital, Yilan, Taiwan

**Keywords:** Physical fitness, Falls, Geriatric population, Grip strength, Single-leg standing

## Abstract

**Background:**

As the community-dwelling population ages in Taiwan, concerns regarding long-term care have grown more urgent. Physical fitness plays a key role in enabling community-dwelling older adults to independently complete daily tasks and avoid falling accidents. However, the effect of physical fitness on falls and other fitness-related factors remains poorly understood.

**Methods:**

In this retrospective cross-sectional study, 2130 community-dwelling older adults were recruited from a rural region of Taiwan. Each of these participants completed a demographics interview and frailty questionnaire and reported their history of falls. We evaluated each participant’s height and body weight measurements, calf circumference, bone mass density, and results on the grip strength, single-leg standing, chair sit-and-reach, 8-ft up-and-go, 30-second chair stand, 2-minute step, 30-second arm curl, 6-m walk, and back scratch tests to determine their overall physical fitness, which consisted of their body composition, muscular strength and endurance, flexibility, and cardiopulmonary fitness.

**Results:**

The prevalence of falls in the preceding year among the older adults surveyed was 20.8%, and the resultant hospitalization rate was 10.9%. The older adults who were more physically active in the past week, had regular exercise habits, lived with family, and had no history of hospitalization due to falls, exhibited greater performance on the physical fitness tests. Three time fallers exhibited lower levels of overall physical fitness than did those who had not fallen. The nonfallers outperformed the fallers in grip strength (participants who had not fallen and those who had fallen once, twice, or three times in the preceding year: 24.66 ± 0.19 vs. 23.66 ± 0.35 vs. 20.62 ± 0.71 vs. 22.20 ± 0.90 kg) and single-leg standing duration (19.38 ± 0.39 vs. 16.33 ± 0.78 vs. 13.95 ± 1.67 vs. 12.34 ± 1.82 seconds).

**Conclusions:**

Exercise habits, living status, hospitalization due to falls, and amount of exercise were all associated with physical fitness in community-dwelling older adults. The results of all of the assessments indicated that the participants who had fallen three times exhibited lower levels of physical fitness than did those who had not fallen in the previous year. Physical measurements, including grip strength and single-leg standing duration, are associated with an individual’s risk of falling, which indicates that they should be considered in the development of geriatric physical fitness and fall-prevention programs.

## Introduction

Physical fitness is a dynamic condition of energy and vitality that allows people to accomplish everyday chores, engage in active leisure, and deal with unforeseen situations without becoming exhausted [1]. Various characteristics, including body size, maturity, time spent exercising, race, and family wealth, may affect an individual’s physical fitness [2]. As the community’s population ages in Taiwan, concerns regarding long-term care have grown in urgency, necessitating an analysis of contributing factors for physical fitness.

Falls are prevalent and problematic events for older adults and are the leading cause of fatal and nonfatal injuries in adults over 65 years old [3, 4]. In the United States, approximately 29.0 million falls occurred in the past year, and 28.7% of older persons have experienced falls, with 37.5% of these requiring medical care or experiencing limitations in completing daily tasks [5]. In Taiwan, 15.1% of older adults have fallen at any point in the preceding year [6], and falling is particularly prevalent among female older adults [6, 7]. As an individual ages, their risk of falling increases for various reasons related to dysfunctional aging processes, including impaired balance and gait patterns, poor vision, arthritis, cognitive decline, and affective disorders [8, 9]. Fall-related injuries, particularly hip and other fractures and head injuries, have the most severe consequences for community-dwelling older adults and can exert a tremendous effect on their quality of life [10]. From the perspective of preventive medicine and rehabilitation, identifying modifiable risk factor and tailoring treatments are essential to reducing the rate of falls and addressing the resultant problems.

Physically active individuals may exhibit a decreased risk of falls and fractures because of their higher levels of physical fitness [11, 12], and sedentary older adults with poor physical fitness must exert greater effort to execute activities necessary for independent living, which increases their risk of falling [13]. Therefore, physical fitness assessments may be viable screening tools for identifying individuals at a high risk of falling to promote healthy aging. A previous study involving community-dwelling older men revealed that grip strength and 6-m walk tests could be used to distinguish individuals at a high risk of falling and of sustaining fractures from those at a low risk [14]. However, an individual’s physical fitness consists of their strength, balance, agility, flexibility, and aerobic fitness, and is necessary to enable the performance of normal daily activities. Moreover, falls and fractures can be predicted by muscle strength, balance, and functional ability [15]. Therefore, physical fitness–related factors other than grip strength and walking speed may also be correlated with falling risk. The results of a survey conducted using self-administered questionnaires revealed that fallers were less active than nonfallers and that physical inactivity was linked to a higher risk of falling and fear of falling [16, 17]. However, questionnaire-based examinations may reflect recall bias. Hence, studies employing thorough physical fitness evaluations can avoid the limitations. Although insufficient physical activity may result in unfavorable outcomes, overtraining may also cause performance issues or increase the risk of falling in older adults because some older adults may have unstable strides or weak physical abilities. Thus, individuals’ physical fitness test results must be systematically analyzed and monitored to enable physicians to improve the poor aspects of physical fitness in the older adult population by promoting appropriate physical activities and thereby reduce the risk of falling among this population.

Although community-dwelling postmenopausal women at a high risk of falling exhibited the lowest performance on gait speed and balance tests [18], various exercise regimens can help older adults improve their physical fitness, function, and overall health. A pilot study involving older adults with surgically repaired hip fractures reported that after a 2-month combination nutritional and rehabilitative intervention, the participants’ biomarkers of age-related sarcopenia had decreased [19]. According to this molecular approach, rehabilitation may enhance the muscle strength and physical performance of older adults, thereby minimizing their risk of falls. Furthermore, by referencing comprehensive analyses of personal features conducted by physicians, experts may develop fall-prevention strategies for older adults and encourage older adults to adopt healthy lifestyles that incorporate organized exercise and dietary supplements to avoid compromising their levels of physical activity due to exhaustion [20].

Despite the availability of extensive research on older adults’ body composition, muscular strength and endurance, flexibility, and cardiovascular fitness, the substantial interindividual variability in physical fitness–related factors affecting individuals’ risk of falling remains a concern. Few large-scale studies have evaluated the effects of physical fitness–related factors on the risk of falling among community-dwelling older adults. The strength of this study is based on its use of a large-scale database. Our findings can serve as references for health care practitioners in planning tailored health promotion programs that incorporate physical activity and dietary assistance to help older adults adopt healthy lifestyles, which may allow them to accomplish daily tasks without growing weary and thereby lower their risk of falling. Regarding rehabilitation, our results suggest that clinicians and therapists can play a key role in implementing fall-prevention strategies by screening older adults for the risk of falling through physical testing in outpatient clinics and modifying a variety of physical fitness–related characteristics. Furthermore, our findings encourage doctors to pay attention not only to diseases but also to older adults’ overall physical fitness and functioning. This community-based study investigated the relationships between physical fitness and demographic variables such as amount of exercise, exercise habits, living alone, and hospitalization due to a fall and identified physical fitness characteristics that may be modified to mitigate the risk of falling among community-dwelling older adults.

## Methods

### Study design and data collection

This was a retrospective cross-sectional study that analyzed the data of community-dwelling older adults who underwent a community health promotion program conducted by the Center of Community Medicine at National Yang Ming Chiao Tung University Hospital between July 10, 2017, and December 25, 2019. This study was approved by the Human Subject Research Ethics Committee of National Yang Ming Chiao Tung University Hospital (IRB No. 2020A012). All procedures were performed in accordance with the declaration of Helsinki’ statement and relevant guidelines. Older adults from communities in Yilan County, Taiwan. were invited to participate. A total of 2878 potential participants were invited to the Center for Health Promotion in Yilan County, Taiwan, and were screened for eligibility. During the recruiting process, the researchers evaluated the participants’ health. All the eligible community-dwelling individuals had passed their initial assessments and were free of serious acute disorders, cognitive impairment, and functional disability at the time of the trial. The participants’ data were collected by qualified therapists and physical fitness coaches with the help of trained hospital volunteers. The participants were required to meet the following inclusion criteria: 1) an age of at least 65 years old and residence in Yilan County, 2) a desire to learn about one’s own physical fitness, and 3) no severe medical problems and the ability to participate in the health promotion program. The exclusion criteria were as follows: 1) severe neurological impairments, 2) substantial cognitive impairments, 3) recent traumatic events, 4) acute illnesses, such as unstable cardiac dysrhythmia, sepsis, or unstable vital signs, and 5) total dependence on caretakers to accomplish daily activities.

Structured questionnaires were administered to all the participants during face-to-face interviews, and the patients’ data—including demographics, medical conditions, fall history, living status, exercise habits, and frailty questionnaire responses [21, 22]—were accessed. Written informed consent was obtained from each participant. Each participant’s physical fitness, including their body composition, muscular strength and endurance, flexibility, and cardiopulmonary fitness, were assessed through height and body weight (Biospace Body Analyser, InBody 570, Biospace CO, Cheonen-si, Korea) and calf circumference and bone mass density (Medilink Ultrasonic Bonedensitometer, Pegasus, Medilink SARL, Mauguio, France) measurements, a hand grip strength test (Digital Hand Dynamometer, TTM 110D, HATAS, Osaka, Japan), a single-leg standing test (Acutek, SHM 15), a chair sit-and-reach test (Acutek, SHM11), an 8-ft up-and-go test (Acutek, SHM13), a 30-second chair stand test (Acutek, SHM 12), a 2-minute step test (Acutek, SHM14), a 30-second arm curl test (Acutek, SHM16), a 6-m walk test, and a back scratch test conducted in accordance with the guidelines of the Sports Department of the Ministry of Education [23, 24]. To improve the efficiency and precision of the tests, we employed a sensor-based assistive system (Acutek Fitness Sencare System, SHM11-16, Acutek, New Taipei City, Taiwan). Each participant’s maximum hand grip strength in their dominant hand was recorded. The amount of exercise performed in the previous week was calculated as each participant’s caloric consumption as reported on their frailty questionnaire [21, 22].

### Statistical analysis

Descriptive statistics are presented herein as numbers of cases and percentages. SPSS 22.00 for Windows (IBM, SPSS, statistics 22) was used to conduct all statistical analyses. Continuous variables are expressed as means ± standard deviations. The threshold for significance was established at *p* < 0.05. Pearson’s correlation coefficients were used to examine the relationships between exercise quantity and physical fitness measures. To identify any significant differences between groups, an independent-samples *t* test was performed (participants with or without exercise habits, who did or did not live alone, and who had or had not been hospitalized due to a fall). The dependent variables were also compared using one-way ANOVA to determine whether any significant differences could be identified among the four groups (participants who had not fallen and those who had fallen once, twice, or three times in the preceding year). The least significant difference or Tamhane test was used for post hoc examination of mean differences.

## Results

A total of 2878 persons were screened for eligibility. Of these, 727 were ineligible for this study (691 under the age of 65, 28 people of unknown age, and 8 who were not residents of Yilan County), and 21 met the exclusion criteria (risk to completing the physical fitness assessments or missing data). Ultimately, 2130 people were recruited for the study.

Among the 2130 participants included in the study, 547 (25.7%) were men and 1583 (74.3%) were women. The mean age of the participants was 74.83 ± 6.54 years. According to the demographic data, 7.2% of the participants had regular exercise habits, and 14.9% lived alone. 20.8% had fallen and 10.9% had been hospitalized due to a fall at any point in the previous year. The frequency of falls was as follows: 2.6% had fallen 3 times, 3.0% had fallen twice, 15.2% had fallen once, and 68.2% had not fallen in the past year. Because of inability to respond due to compromised bodily functions, and low-quality (incomplete, implausible, or inconsistent) responses, the nonresponse rate was 11%. Table 1 lists the demographic and clinical characteristics of all the participants.

**Table 1:** Participant demographics (*N* = 2130)

Item	n	%
Gender		
Female	1583	74.3
Male	547	25.7
Age (years)		
65–69	607	28.5
70–74	518	24.3
75–79	533	25
80–84	317	14.9
85–89	129	6
90 or older	26	1.2
Exercise habit		
No habits	1874	88
With habits	153	7.2
Living status		
Alone	318	14.9
With family	1641	77
Hospitalized due to a fall in the preceding year		
Yes	233	10.9
No	1397	65.6
Falls in the preceding year		
0	1453	68.2
1	323	15.2
2	64	3
3 or more	56	2.6

### Correlation analysis of physical fitness

**Fig. 1:**
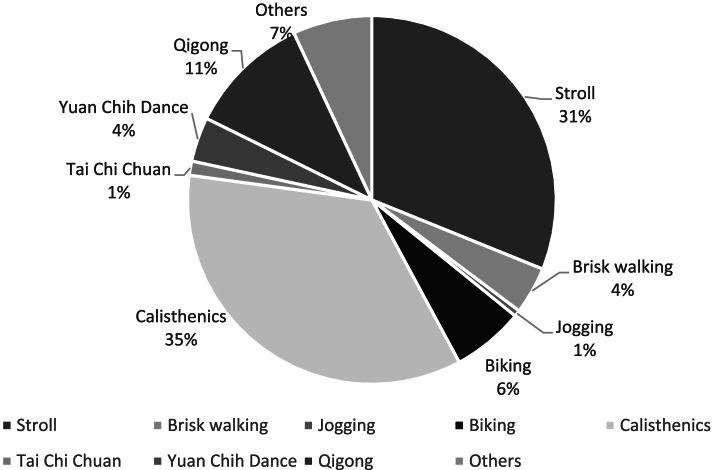
Types of exercise performed by community-dwelling older adults (*N* = 1889)

Figure 1 illustrates the types of physical activities undertaken by community-dwelling older adults. The effects of independent factors on the results of each physical fitness test were examined using Pearson’s correlation coefficient. The total caloric consumption in the week prior to testing, which reflected the amount of exercise performed, was correlated with the physical fitness measures; in other words, physical fitness parameters—namely the hand grip strength (correlation coefficient/*p*-value = r/*p* = 0.095/0.000), 8-ft up-and-go (r/*p* = − 0.095/0.000), 30-second chair stand (r/*p* = 0.088/0.000), 30-second arm curl (r/*p* = 0.112/0.000), 6-m walk test (r/*p* = − 0.082/0.000) test results and bone mass density (r/*p* = 0.085/0.006)—were associated with the amount of exercise performed in the preceding week. These findings are presented in Table 2.

**Table 2:** Pearson’s correlation coefficients for amount of exercise and physical fitness metrics

Physical fitness (unit)	Amount of exercise (total caloric consumption/Kcal)		
	n	*r*	*p* value
Hand grip strength (kg)	2127	0.095^b^	0.000
Single-leg standing (s)	2082	−0.041	0.060
Chair sit-and-reach (cm)	2100	0.005	0.831
8-ft up-and-go (s)	2129	−0.095^a^	0.000
30-second chair stand (number of repeats)	2115	0.088^a^	0.000
2-minute step (number of steps)	2068	0.032	0.147
30-second arm curl (number of repeats)	2118	0.112^a^	0.000
6-m walk test (s)	2127	−0.082^a^	0.000
Back scratch (cm)	2068	0.034	0.122
Calf circumference (cm)	1020	0.038	0.220
Bone mass density (T-score)	1020	0.085^a^	0.006

*r*: correlation coefficient, ^a^ correlation is significant at the 0.01 level and ^b^ at the 0.05 level (two-tailed)

The results of the independent-samples *t* test used to compare the physical fitness of the participants with or without exercise habits, who did or did not live alone, and who had or had not been hospitalized due to a fall are displayed in Table 3. Although the differences in grip strength, calf circumference, and bone mass density were negligible, the participants with exercise habits scored higher on all the physical fitness tests than did those without exercise habits. Those who lived with family exhibited greater physical fitness than did those who lived alone, particularly accordingly to the results of the grip strength, single-leg standing, 8-ft up-and-go, 30-second chair stand, 30-second arm curl, and 6-m walk tests. The participants who had been admitted to the hospital after falling received lower scores on the 8-ft up-and-go, 30-second chair stand, 6-m walk, and back scratch tests than did those who had not been hospitalized due to a fall (9.23 seconds, 14.9 repeats, 6.47 seconds, and − 11.23 cm vs. 8.50 seconds, 16.2 repeats, 5.63 seconds, and − 8.9 cm, respectively). In summary, the older adults who exercised regularly, lived with family, and had no history of hospitalization due to falls exhibited greater physical fitness.

**Table 3:** Comparison of physical fitness between elders with or without exercise habit, who are or are not living alone, and who have or have not been hospitalized due to a fall

Physical fitness measurement	Exercise habit (mean ± SE)			Living status (mean ± SE)			Hospitalized due to a fall (mean ± SE)		
	With (*N* = 1873)	Without (*N* = 153)	*p* value	Alone (*N* = 318)	With family (*N* = 1641)	*p* value	Ever (*N* = 233)	No (*N* = 1396)	*p* value
Height (cm)	154.12 ± 0.17	154.21 ± 0.65	0.873	152.44 ± 0.38	154.46 ± 0.18	0.000*	153.48 ± 0.49	154.36 ± 0.19	0.084
Body weight (kg)	60.18 ± 0.24	61.47 ± 0.9	0.137	59.01 ± 0.55	60.50 ± 0.26	0.018*	59.89 ± 0.71	60.59 ± 0.28	0.336
Hand grip strength (kg)	24.41 ± 0.16	23.58 ± 0.6	0.153	22.81 ± 0.35	24.63 ± 0.17	0.000*	23.62 ± 0.44	24.44 ± 0.18	0.097
Single-leg standing (s)	18.73 ± 0.34	15.93 ± 1.04	0.012*	15.37 ± 0.81	18.96 ± 0.37	0.000*	18.50 ± 1.09	18.17 ± 0.39	0.766
Chair sit-and-reach (cm)	5.10 ± 0.24	2.04 ± 0.77	0.000*	4.15 ± 0.58	4.90 ± 0.25	0.233	5.16 ± 0.68	4.95 ± 0.27	0.774
8-ft up-and-go (s)	8.38 ± 0.81	10.04 ± 0.53	0.002*	9.10 ± 0.22	8.47 ± 0.10	0.008*	9.23 ± 0.28	8.50 ± 0.10	0.009*
30-second chair stand (number of repeats)	16.2 ± 0.1	14.2 ± 0.4	0.000*	15.3 ± 0.3	16.1 ± 0.1	0.014*	14.9 ± 0.3	16.2 ± 0.1	0.001*
2-minute step (number of steps)	109.4 ± 0.5	104.5 ± 1.8	0.005*	106.8 ± 1.2	109.0 ± 0.5	0.080	109.8 ± 1.4	108.3 ± 0.6	0.302
30-second arm curl (number of repeats)	18.5 ± 0.1	16.9 ± 0.4	0.000*	17.7 ± 0.2	18.4 ± 0.1	0.006*	17.8 ± 0.3	18.4 ± 0.1	0.063
6-m walk test (s)	5.63 ± 0.05	6.59 ± 0.37	0.012*	6.03 ± 0.13	5.67 ± 0.06	0.020*	6.47 ± 0.21	5.63 ± 0.07	0.000*
Back scratch (cm)	−9.01 ± 0.31	− 12.93 ± 1.03	0.000*	−9.89 ± 0.72	−9.43 ± 0.33	0.575	− 11.23 ± 0.88	−8.90 ± 0.35	0.013*
Calf circumference (cm)	34.03 ± 0.11	33.61 ± 0.47	0.352	33.57 ± 0.27	34.07 ± 0.11	0.079	33.41 ± 0.52	34.17 ± 0.12	0.095
Bone mass density (T-score)	−1.072 ± 0.039	− 1.016 ± 0.176	0.736	−1.175 ± 0.978	− 1.073 ± 0.043	0.349	−1.161 ± 0.156	−1.052 ± 0.044	0.502

*p < 0.05, independent *t* test

### Effect of physical fitness on risk of falling

The difference in physical fitness among community-dwelling older adults who had fallen 0 to 3 times in the preceding year was investigated through one-way ANOVA. The participants’ physical fitness assessment results were compared with their numbers of falling incidents in the previous year. The participants who had fallen 3 times in the previous year exhibited lower physical fitness on all the tests than those who had not fallen. The participants who had fallen three times in the preceding year exhibited lower performance on the 8-ft up-and-go, 30-second chair stand, 30-second arm curl, and back scratch tests than did those who had fallen only once (10.38 ± 0.59 vs. 8.68 ± 0.19 seconds, 13.0 ± 0.7 vs. 15.7 ± 0.3 repeats, 17.0 ± 0.6 vs. 18.4 ± 0.2 repeats, and − 16.76 ± 1.97 vs. − 10.05 ± 0.77 cm, respectively). The participants who had fallen twice received lower average scores on the grip strength, single-leg standing, 30-second chair stand, and 30-second arm curl tests than did those who had not fallen in the preceding year (20.62 ± 0.71 vs. 24.66 ± 0.19 kg, 13.95 ± 1.67 vs. 19.38 ± 0.39 seconds, 14.6 ± 0.7 vs. 16.3 ± 0.1 repeats, and 16.7 ± 0.5 vs. 18.5 ± 0.1 repeats, respectively). The participants who had fallen twice received lower average scores on the grip strength and 30-second arm curl test than did those who had fallen once. Furthermore, the participants who had not fallen received higher scores on the grip strength (24.66 ± 0.19 kg) and single-leg standing (19.38 ± 0.39 seconds) test than did those who had fallen at all during the previous year. Table 4 presents the effects of physical fitness on the frequency of falls in community-dwelling older adults.

**Table 4:** Differences in physical fitness among community-dwelling older adults who had fallen 0 to 3 times in the previous year

Physical fitness measurement (unit / mean ± SE)	Falls				*p* value					
	0	1	2	3	0 vs. 1	0 vs. 2	0 vs. 3	1 vs. 2	1 vs. 3	2 vs. 3
Height (cm)	154.32 ± 0.19	153.63 ± 0.39	152.35 ± 0.80	153.14 ± 0.96	–	–	–	–	–	–
Body weight (kg)	60.28 ± 0.27	60.62 ± 0.62	57.86 ± 1.01	60.66 ± 1.42	–	–	–	–	–	–
Hand grip strength (kg)	24.66 ± 0.19	23.66 ± 0.35	20.62 ± 0.71	22.20 ± 0.90	0.018*	0.000*	0.008*	0.001*	0.142	0.207
Single-leg standing (s)	19.38 ± 0.39	16.33 ± 0.78	13.95 ± 1.67	12.34 ± 1.82	0.003*	0.014*	0.002*	0.736	0.256	0.987
Chair sit-and-reach (cm)	4.86 ± 0.28	5.10 ± 0.52	7.27 ± 1.23	3.15 ± 1.37	–	–	–	–	–	–
8-ft up-and-go (s)	8.34 ± 0.10	8.68 ± 0.19	9.92 ± 0.67	10.38 ± 0.59	0.549	0.136	0.007*	0.402	0.047*	0.996
30-second chair stand (number of repeats)	16.3 ± 0.1	15.7 ± 0.3	14.6 ± 0.7	13.0 ± 0.7	0.068	0.010*	.000*	0.107	0.000*	0.101
2-minute step (number of steps)	109.4 ± 0.5	108.1 ± 1.1	101.6 ± 3.2	101.2 ± 3.9	–	–	–	–	–	–
30-second arm curl (number of repeats)	18.5 ± 0.1	18.4 ± 0.2	16.7 ± 0.5	17.0 ± 0.6	0.690	0.002*	0.015*	0.005*	0.034*	0.670
6-m walk test (s)	5.59 ± 0.07	5.90 ± 0.14	6.72 ± 0.49	6.83 ± 0.35	0.249	0.144	0.005*	0.506	0.087	1.000
Back scratch (cm)	−8.71 ± 0.35	−10.05 ± 0.77	−11.04 ± 1.35	−16.76 ± 1.97	0.509	0.468	0.001*	0.989	0.013*	0.106
Calf circumference (cm)	34.06 ± 0.12	34.20 ± 0.27	33.10 ± 0.57	33.14 ± 0.74	–	–	–	–	–	–
Bone mass density (T-score)	−1.081 ± 0.046	−1.098 ± 0.092	−0.957 ± 0.204	−1.290 ± 0.225	–	–	–	–	–	–

*p < 0.05; One-way ANOVA was used to identify significant differences (*p* < 0.05) in the dependent variables among the four groups. Post hoc analysis of the mean differences was performed using the least significant difference or Tamhane test

**Fig. 2:**
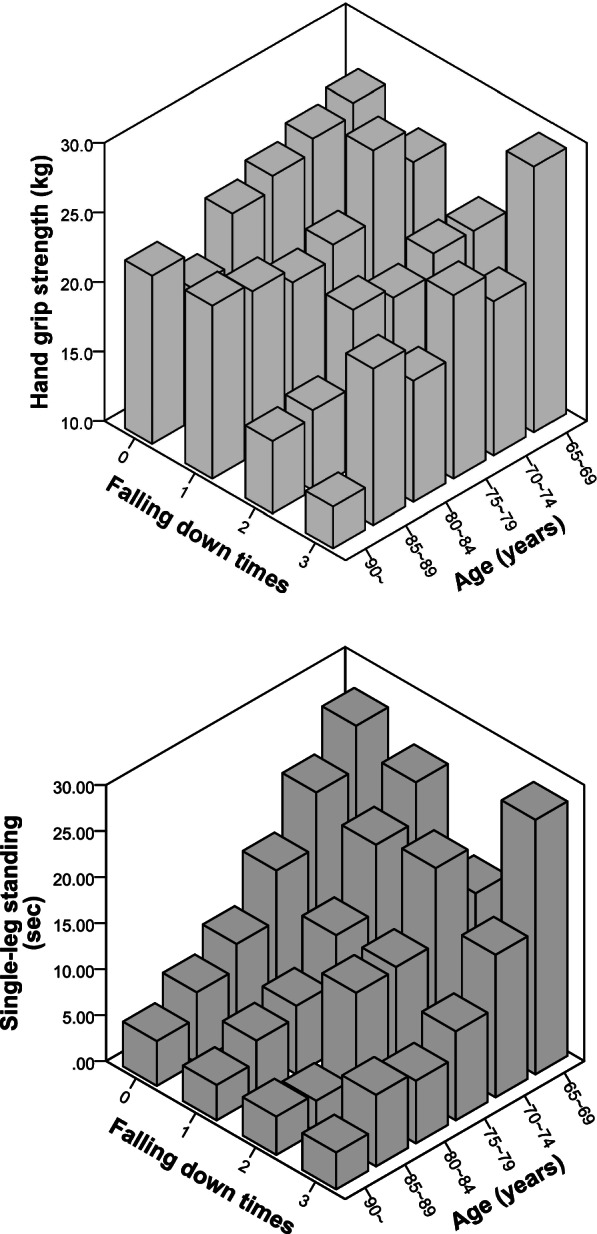
Three-dimensional histogram of participants’ ages, frequency of falling, and scores on two physical fitness tests (grip strength and single-leg standing)

As indicated in Table 4, we stratified the participants by age, frequency of falls, and two physical fitness tests (grip strength and single-leg standing). In the groups of participants aged 80–84 years and ≥ 90 years, poor grip strength was associated with a higher frequency of falling. In the groups of participants aged 70–74 years, 75–79 years, and ≥ 90 years, a shorter duration of single-leg standing was associated with a higher frequency of falling. A three-dimensional histogram of the data is presented in Fig. 2.

## Discussion

In this study, the physical fitness of community-dwelling older adults in rural Taiwan was associated with several characteristics, including the amount of exercise performed in the preceding week, exercise habits, living status, and history of hospitalization due to falls. Because few community-dwelling older men participate in community activities, most of the older adults recruited in this study were women. This may be attributable to the fact that in Taiwan’s rural areas, most elderly men who had previously worked as laborers or farmers often take labor as exercise. Furthermore, certain community programs established by women are uninteresting to older men. In traditional Taiwanese societies, men are dominant in families and frequently encounter barriers to participate in community activities, particularly if they have recently retired from their jobs. These remind the healthcare professionals that gender differences must be considered while organizing community activities. The older individuals who exercised regularly, lived with family, and had no history of hospitalization due to falls generally exhibited greater physical fitness than those who did not meet these criteria. Furthermore, older adults who had not fallen in the previous year achieved higher scores on the grip strength and single-leg standing tests than did those who had fallen, indicating that these tests might be used as screening tools for a high risk of falling. Although falls are common, nearly half of older adults who fall do not inform their health care providers. However, falls are not unavoidable occurrences for older adults. The analysis of physical fitness–related variables in this study has implications for public health practice. Using our results, health-care workers in preventive medicine and rehabilitation institutions may implement fall-prevention methods, such as assessing and controlling changeable factors associated with physical fitness, screening older people for fall risk through physical testing, and recommending optimal exercise programs to address individual weaknesses in physical fitness.

Given that the average life expectancy is increasing, healthy aging—which involves minimizing functional deterioration and maintaining one’s quality of life—is a priority. The adoption of a healthy lifestyle that incorporates physical activity and adequate nutrition can play a key role in the maintenance of physical fitness and functions associated with successful aging. One study reported that a 2-month rehabilitative program combined with nutritional consultation exerted a positive effect on functional abilities of patients aged 65 years and over with hip fractures [25]. Notably, incorporating essential amino acids into the diet of patients with sarcopenic hip fractures resulted in considerable improvements, which underscores the efficacy of a planned exercise program paired with nutritional intervention [25]. Furthermore, physical activity and dietary supplements have been demonstrated to affect molecular pathways in older adults with osteoarthritis, which suggests that this convergent strategy might help attenuate the complicated pathological characteristics of disease [26]. Although exercise programs may benefit an individual’s functional performance, older adults are less inclined to participate in such programs. The authors recommend that nutritional supplements be incorporated into intervention designs to encourage older adults’ participation in exercise programs [27]. Overall, a combination of physical activity and adequate nutrition benefits older adults, and the establishment of healthy lifestyles should be prioritized in any health promotion program.

Rosengren et al. reported that among community-dwelling adults aged 65 years or older, the grip strength and 6-m walk tests could be used to distinguish those who had sustained fractures from falling from those who had not fallen or who had fallen without fracture [14]. Our study focused on community-dwelling older adults of either gender who were willing to participate in our program and complete all the physical examinations. Those who passed the initial screening and met the inclusion criteria had adequate functional capacities and therefore exhibited the potential to benefit from physical activity–focused interventions designed to address individual weaknesses identified using the physical fitness assessments. Several physical fitness tests, including grip strength [28], chair stand [28], single-leg standing [28], timed up-and-go [28, 29], 10-m walk [29], and 6-minute walk [28, 29] tests, have been used as the screening instruments for fall risk and daily function in the older adults with frailty. An individual’s muscular strength, balance, and functional skills can be used to predict their risk of falls and fractures [15]. Six-meter walk and grip strength tests are insufficient for evaluating an individual’s risk of falling occurrences, and complete physical evaluations are often required to recommend appropriate exercise regimens. Our study evaluated the relationships between several key elements of physical fitness and the occurrence of falls in the previous year and may therefore serve as a reference for physicians in evaluating individuals and tracking impaired motor skills. Unsurprisingly, the participants who had fallen three times in the previous year exhibited poorer performance than did those who had not fallen on all the physical fitness tests and even received significantly lower scores on some of the test than did the participants who had fallen once. Significant differences in performance on the grip strength and single-leg standing tests were identified between the participants who had fallen and those who had not fallen in the previous year. Nonetheless, according to our data, the 6-m walk test can only differentiate participants who had fallen three times fallers from those who had not fallen in the preceding year and lacks the sensitivity to distinguish participants who had not fallen in the preceding year from those who had fallen fewer than three times. The setting in which the test was administered, as well as the diminished physical functioning and mental inattention of the participants, particularly in those with frailty, influenced the results of the 6-m walk test. Fundamental components of physical fitness, such as static standing balance and overall muscular strength, as measured by the single-stand test and grip strength, respectively, were revealed to be the primary predictors of falls in our study. Although falls can be caused by various factors, older adults may be at a higher risk of falling if they have muscular weakness or static balance dysfunction.

Grip strength can be used to diagnose sarcopenia and frailty [22, 30] and is associated with appendicular skeletal muscle mass in older Chinese adults [31]. Previous studies on geriatric populations have revealed that muscle strength testing can predict functional prognoses, such as falls and ambulatory abilities, in older adults to a higher extent than can time-consuming and high-cost muscle mass examinations [32, 33]. These findings suggest that grip strength tests can be used as screening tools to evaluate risk of falling in older adults and are consistent with a recent study that determined that increasing exercise duration and grip strength lowered an individual’s chance of falling [6]. According to a Taiwanese community study involving older adults who resided in the same region from August 2013 to February 2016, the average grip strengths of participants who had not fallen and who had fallen were 20.7 kg and 17.6 kg, respectively [6]. The participants in our study exhibited higher grip strength than did those in other studies (24.66 kg in those who had not fallen in the preceding year and 23.66 kg, 20.62 kg, and 22.20 kg in those who had fallen once, twice, or three times, respectively). This might be explained by the fact that the data were obtained using a different approach. Rather than collecting data from broad geriatric populations by conducting home visits as in prior studies [6], our team collected data from more active older adults who were able to participate in the program and complete all physical fitness tests at the Center for Health Promotion.

One retrospective study reported that the participants who had fallen during 1 year exhibited lower performance in the single-leg standing test than did those who had not fallen, and the difference remained significant when the participants’ performance levels were dichotomized using a cutoff value [28]. However, a comprehensive evaluation of the three types of screening tests for falls in older adults revealed that the single-leg standing test had a sensitivity and specificity of 0.51 and 0.61, respectively, and 0.33 and 0.712, respectively, for recurrent falls [34]. According to the authors of this evaluation, all the tests exhibited low diagnostic accuracy as well as inconsistency in predicting future falls in older adult populations [34]. Our large-scale study revealed that participants who had not fallen in the preceding year had a higher single-leg standing time (19.38 ± 0.39 seconds) than did those who had fallen, which supports the predictive value of the test for risk of falling in older adults.

Community-dwelling older adults often experience difficulty accessing health care and transportation as well as environmental restrictions that impede physical exercise. Moreover, family disintegration and the migration and emigration of young adults has led to an increase in the number of older adult citizens living alone in Taiwan’s rural areas, and these older adults are more likely to have poor health, to rely on others, and to be hospitalized or institutionalized [35, 36]. Maintaining an adequate degree of physical fitness is one of the pillars of successful aging, and it is especially crucial for older adults who live alone in rural regions. Hence, experts must first identify the variables related to an individual’s physical fitness level before developing an appropriate physical fitness training program. A previous study reported that body size, maturity, time spent exercising, race, and family affluence all affect the physical fitness of children and adolescents [2]. Our findings reveal that living alone is related to poor physical fitness in older adults, and almost all of the extant literature has indicated that social isolation and loneliness have negative consequences on physical and mental health in later life [37]. We also identified numerous other determining factors of physical fitness in community-dwelling older adults, including regular exercise habits and amount of physical activity and a history of hospitalization due to falls. Some of the factors identified, such as regular exercise habits, living status, and amount of physical activity, can be modified to promote an individual’s physical fitness and should therefore be considered by clinical practitioners when developing health promotion interventions. Engaging in exercise, establishing a supporting system, and maintaining physical activity as one ages may enhance the physical fitness of older adults.

The connections between various physical fitness metrics and the amount of exercise performed in the previous week are unsurprising. Our findings revealed a relationship among the results of the hand grip strength, 8-ft up-and-go, 30-second chair stand, 30-second arm curl, and 6-m walk tests, which were used to assess the participants’ total muscular strength, agility and dynamic balance, lower limb endurance, upper limb endurance, and functional capabilities. The outcomes were consistent with what we had expected. However, the results of the 2-minute step test, which measures cardiorespiratory fitness, were unrelated to the amount of physical exercise performed in the previous week. The distinction between exercise and cardiovascular training might explain this discrepancy. Exercise may enhance or maintain an individual’s physical fitness by preserving their muscular strength and other fitness characteristics and is defined as deliberate, organized, repeated, and purposeful movement [38]. Cardiovascular training is any exercise that works the heart and vascular systems to increase the heart’s ability to pump blood and supply oxygen to the body’s tissues [39]. If older individuals want to enhance their cardiopulmonary fitness, they must engage in cardiovascular training of a sufficient intensity.

Although this study provided meaningful findings regarding multiple outcomes and employed a large-scale database, it still has several limitations. First, we employed a retrospective cross-sectional study design, which has inherent weaknesses, including the inability to measure incidence and identify causal relationships. Therefore, more long-term studies are warranted. Second, the participants in this study were active older adults with high levels of physical performance and daily functioning. Hence, whether our findings are applicable to older adults with frailty who were unable to participate in our study is unclear. Third, certain demographic data were collected using a self-administered survey, which is prone to recall bias. Fourth, the study’s clinical implications for specific populations are limited due to the lack of data regarding the participants’ specific conditions. Finally, to assess the participants’ risk of falling, standard measures such as the Berg Balance Scale and the Tinetti scale were utilized. However, the use of these instruments requires the participation of medical experts or qualified testers and involves a series of activities that takes approximately 15 minutes to complete for each participant. Most of the older adults who participated in our study traveled to our center by a shuttlebus supplied by the county government. Due to time restrictions, we did not record some characteristics such as daily medications, comorbidities, and fall risk assessment scale scores.

## Conclusions

In this study, we evaluated the relationships between physical fitness and regular exercise habits, living situations, hospitalization due to falls, and quantity of exercise. Physical fitness is associated with the risk of falling among community-dwelling older adults. On all the tests, the participants who had fallen three times in the previous year exhibited lower physical fitness than did those who had not fallen. The participants who had not fallen exhibited higher grip strength and single-leg standing ability than did those who had fallen in the previous year, indicating that these two tests may be used to develop personalized physical fitness programs and fall-prevention methods for older adults.

## Data Availability

The datasets generated and/or analyzed during the current study are not publicly available due to privacy but are available from the corresponding author on reasonable request.
